# Early prophylactic low-dose hydrocortisone for bronchopulmonary dysplasia prevention in extremely preterm infants: a systematic review and meta-analysis

**DOI:** 10.3389/fped.2026.1934115

**Published:** 2026-10-26

**Authors:** Lamar Abu Shehadeh, Tala Adel Al Ghamdi, Rahaf Atiah Alzahrani, Bayan Yahya Mushari, Layan Abdulrahman Alotaibi, Lara Sadik, Omar Mohammad Kheder, Turki Hagr, Bedah Mohammed Alnawfal

**Affiliations:** 1 College of Medicine, Alfaisal University, Riyadh, Saudi Arabia; 2 College of Medicine, Imam Abdulrahman Bin Faisal University, Dammam, Saudi Arabia; 3 College of Medicine, King Saud bin Abdulaziz University for Health Sciences (KSAU-HS), Jeddah, Saudi Arabia; 4 Saudi Board in Pediatric, Riyadh, Saudi Arabia; 5 College of Medicine, Almaarefa University, Riyadh, Saudi Arabia; 6 Department of Pediatric, Prince Mohammed Bin Abdulaziz Hospital, Madinah, Saudi Arabia; 7 King Saud Medical City, Riyadh, Saudi Arabia; 8 Department of Pediatrics Hematology and Oncology, King Saud Medical City, Riyadh, Saudi Arabia

**Keywords:** bronchopulmonary dysplasia, corticosteroids, extremely preterm infants, hydrocortisone, meta-analysis, neonatal outcomes

## Abstract

**Background:**

Early prophylactic low-dose hydrocortisone has been proposed to reduce inflammation and to compensate for relative adrenal insufficiency in extremely preterm infants. Its overall balance of benefit and risk remains uncertain.

**Objective:**

To evaluate whether hydrocortisone initiated within the first 24–48 h after birth reduces bronchopulmonary dysplasia (BPD) and mortality in infants born before 30 weeks' gestation.

**Methods:**

This systematic review and meta-analysis followed PRISMA 2020 guidance and was registered in PROSPERO. PubMed, Cochrane CENTRAL, Scopus, and Web of Science were searched from inception to May 2026. Eligible studies enrolled infants born before 30 weeks' gestation who received early prophylactic systemic hydrocortisone at a low dose. Low dose was defined as a starting dose of no more than 2.0 mg/kg/day, begun within 24–48 h of birth. The comparator was placebo or no hydrocortisone. Randomized and quasi-randomized trials, their follow-up reports, and comparative observational studies were eligible. Random-effects meta-analyses were performed using risk ratios (RRs) or mean differences with 95% confidence intervals (CIs).

**Results:**

Fourteen studies with 3,416 infants were included. Early hydrocortisone did not significantly improve survival without BPD at 36 weeks' postmenstrual age (RR: 1.07, 95% CI: 0.97–1.19, *p* = 0.18). It also did not significantly reduce BPD at 36 weeks (RR: 0.94, 95% CI: 0.85–1.04, *p* = 0.25) or mortality before discharge (RR: 1.07, 95% CI: 0.77–1.49, *p* = 0.69). The duration of respiratory support and the need for oxygen at discharge were similar between groups. Subgroup analyses suggested a possible benefit in infants exposed to chorioamnionitis and in those born before 26 weeks, but these findings were exploratory. Hydrocortisone increased the risk of late-onset sepsis (RR: 1.19, 95% CI: 1.05–1.35, *p* = 0.01). Gastrointestinal perforation, necrotizing enterocolitis, major neonatal morbidities, and the available neurodevelopmental outcomes did not differ significantly between groups.

**Conclusion:**

Current evidence does not support the routine use of early prophylactic hydrocortisone in all extremely preterm infants. Adequately powered trials and individual patient data analyses are needed to identify the subgroups most likely to benefit and to clarify long-term safety.

**Systematic Review Registration:**

https://www.crd.york.ac.uk/prospero/view/CRD420261402317, PROSPERO CRD420261402317.

## 1 Introduction

Extreme preterm birth remains strongly associated with early mortality and chronic respiratory morbidity. This is the case despite advances in antenatal care, surfactant therapy, non-invasive ventilation, nutrition, and neonatal intensive care (1, 2). In these infants, bronchopulmonary dysplasia (BPD) is a common and clinically important complication (3). Its reported prevalence depends on the definition used. Among very preterm or very low birth weight infants, the prevalence is approximately 35% at 28 days and 21% at 36 weeks' postmenstrual age. In extremely low gestational age infants, it may exceed 40% (4).

BPD has a multifactorial pathogenesis. Alveolar and pulmonary vascular development is arrested in the setting of antenatal inflammation, postnatal infection, oxygen exposure, and mechanical ventilation. Immature antioxidant and endocrine responses also contribute (3, 5–7). Early relative adrenal insufficiency may worsen cardiovascular instability and pulmonary inflammation. This provides a rationale for corticosteroid use during this vulnerable period of lung development (8). Systemic corticosteroids have long been studied as a way to reduce pulmonary inflammation and improve respiratory status in preterm infants (9, 10). However, early high-dose dexamethasone has been associated with gastrointestinal, infectious, metabolic, and neurodevelopmental harms (11, 12). Hydrocortisone differs from dexamethasone in potency, mineralocorticoid activity, biologic profile, and dosing (13). Early low-dose hydrocortisone has therefore been proposed as prophylaxis. Its purpose is to compensate for adrenal insufficiency and to limit early inflammatory lung injury. It is not intended as rescue therapy for established disease (14–16).

Evidence from randomized studies suggests that early prophylactic hydrocortisone may improve survival without BPD in extremely preterm infants. In the PREMILOC trial, 523 infants born at 24–27 + 6 weeks' gestation were randomized within 24 h of birth to low-dose hydrocortisone or placebo. Survival without BPD at 36 weeks' postmenstrual age was higher in the hydrocortisone group than in the placebo group (60% vs. 51%) (17). The same trial also raised a safety concern. Among infants born at 24–25 weeks' gestation, late-onset sepsis was more frequent with hydrocortisone than with placebo (40% vs. 23%). In the trial population as a whole, the rate of sepsis did not differ significantly between groups (17). Any benefit of hydrocortisone must therefore be weighed against a possible risk of infection in the most immature infants. Uncertainty also remains about uncommon serious adverse events. One example is spontaneous gastrointestinal perforation, which has been linked to the use of hydrocortisone together with indomethacin for the treatment of patent ductus arteriosus (PDA) (14). In addition, previous studies differ in gestational age, timing, dose, duration, co-interventions, and outcome definitions (15, 18, 19). This systematic review and meta-analysis therefore aimed to evaluate whether hydrocortisone started within 24–48 h of birth reduces BPD and all-cause mortality before discharge in infants born before 30 weeks' gestation.

## 2 Methods

### 2.1 Study design and protocol registration

This systematic review and meta-analysis was conducted in accordance with the Preferred Reporting Items for Systematic Reviews and Meta-Analyses (PRISMA) 2020 statement (20). The methods followed the recommendations of the Cochrane Handbook for Systematic Reviews of Interventions (21). The protocol was registered in PROSPERO before data extraction (CRD420261402317). Deviations from the registered protocol were documented and justified.

### 2.2 Eligibility criteria

Studies were selected using predefined eligibility criteria based on the PICO framework. The population comprised infants who were born before 30 weeks' gestation and were cared for in a neonatal intensive care unit. The intervention was early prophylactic systemic hydrocortisone. Prophylactic use meant that hydrocortisone was given to prevent BPD, not to treat established or evolving disease. Low dose was defined *a priori* as a starting dose of no more than 2.0 mg/kg/day. The total course, including tapering, could not exceed 15 days. Treatment had to start within the first 24–48 h after birth. Regimens with a higher starting dose or a later start were not eligible. The comparator was placebo or no hydrocortisone.

Randomized and quasi-randomized controlled trials and their prespecified follow-up reports were eligible. Comparative observational studies were also eligible, because randomized evidence for this indication is limited. An observational study was included only if it met four conditions. First, the exposure met the definition of early prophylactic low-dose hydrocortisone given above. Second, the study had a concurrent or historical comparator group that did not receive early hydrocortisone and came from the same or an equivalent care setting. Third, outcomes were reported separately for exposed and unexposed infants. Fourth, the study reported at least one prespecified review outcome.

The following were excluded: non-comparative observational studies, case reports, case series, reviews, editorials, conference abstracts without extractable outcome data, animal studies, and pharmacokinetic-only studies. Studies of hydrocortisone as rescue therapy for established or evolving BPD were also excluded. Studies of other corticosteroids were excluded unless they reported hydrocortisone data separately.

No included study used an upper gestational age limit above 30 weeks. Eleven reports set an explicit upper limit of 27, 28, 29, or 30 weeks. The three reports of the Watterberg trial program defined eligibility by birth weight alone (500–999 g). The mean gestational age at enrollment in these three reports was 25.2–25.4 weeks. Four of the eleven reports, from two trial programs, combined an upper limit of 30 weeks with a birth weight ceiling of 1,250 g. A small number of infants born between 30 + 0 and 30 + 6 weeks could therefore have been enrolled in these studies. Their mean gestational age was 26.3–26.8 weeks. No pooled estimate in this review combines infants born above and below 30 weeks. Study-level eligibility criteria are given in Table 1.

**Table 1:** Summary of the included studies.

Author (year)	Study design	Country	Recruitment period	Follow-up	Timing of hydrocortisone initiation	Hydrocortisone dose/duration	Comparator	Participants	Sample size			BPD definition	Primary outcome	Main conclusion
									Total	Intervention	Control			
Baud et al. (2016) (17) PREMILOC	Double-blind, multicenter, placebo-controlled, randomized trial	France; 21 tertiary-care NICUs	May 2008 to Jan 2014	Until 36 wks. PMA	Within first 24 postnatal hours	IV hydrocortisone 1 mg/kg/day divided twice daily for 7 days, then 0.5 mg/kg/day once daily for 3 days	Placebo, IV glucose 5%	Inborn extremely preterm infants delivered at 24 to 27 weeks' gestation	521	255	266	Combined oxygen and ventilation support with a standardized assessment at 36 weeks (plus or minus 3 days) of PMA	Survival without BPD at 36 weeks PMA	Early low-dose hydrocortisone significantly increased survival without BPD at 36 weeks PMA: 60% vs 51%
Baud et al. (2017) (26)	2-year follow-up of Baud (2016) (17)	France	May 2008 to Jan 2014	Median 22 months	Within first 24 postnatal hours	IV hydrocortisone 1 mg/kg/day divided twice daily for 7 days, then 0.5 mg/kg/day once daily for 3 days	Placebo, IV glucose 5%	Surviving PREMILOC infants delivered at 24 to 27 weeks' gestation	379	194	185	NA	Neurodevelopmental impairment at 2 years	Early low-dose hydrocortisone was not associated with worse neurodevelopmental outcomes at 2 years
Bonsante et al. (2007) (27)	Double-blind, randomized, placebo-controlled trial	Italy; 2 NICUs	April 2003 to September 2005	36 wks. PMA; neurodevelopment planned at 2 yrs	Within 48 h of life	IV hydrocortisone 0.5 mg/kg every 12 h for 9 days, then 0.5 mg/kg/day for 3 days	Placebo/normal saline	Mechanically ventilated infants, GA 24–30 wks., birth weight 500–1,249 g, after rescue surfactant	50	25	25	Oxygen dependence at 36 wks. postconceptional age	Survival without supplemental oxygen at 36 wks. PMA.	Hydrocortisone improved O₂-free survival and early circulatory function, with no important short-term adverse effects reported
Efird et al. (2005) (28)	Randomized, double-blind, placebo-controlled pilot trial	USA	May 2000 to May 2002	Until hospital discharge	Within first 3 h of life	IV hydrocortisone 1 mg/kg every 12 h for 2 days, then 0.3 mg/kg every 12 h for 3 days	Normal saline placebo	Inborn ELBW infants; GA >23 to <29 wks.; BW >500 to <1,000 g	34	16	18	Need for oxygen at 36 wks. PMA	Vasopressor treatment for protocol-defined hypotension	Hydrocortisone reduced vasopressor-treated hypotension in the first 2 days
Peltoniemi et al. (2005) (29)	Randomized, blinded, placebo-controlled trial	Finland	August 2002 to Mar 2004	36 wks. PMA; until hospital discharge	Before 36 h of age	IV hydrocortisone: 2.0 mg/kg/day for 2 days, then 1.5 mg/kg/day for 2 days, then 0.75 mg/kg/day for 6 days	Isotonic saline placebo	Infants GA 23 to 30 wks., BW 501–1,250 g, with respiratory failure requiring MV	51	25	26	Need for supplemental oxygen at 36 wks. PMA	Survival without BPD at 36 + 0 wks. PMA	Hydrocortisone did not significantly increase survival without BPD overall, but BPD tended to be lower and PDA was significantly reduced.
Peltoniemi et al. (2009) (30)	2-year follow-up of Peltoniemi (2005) (29)	Finland	August 2002 to Mar 2005	2 years corrected age	Before 36 h of age	IV hydrocortisone: 2.0 mg/kg/day × 2 days, then 1.5 mg/kg/day × 2 days, then 0.75 mg/kg/day × 6 days	Isotonic saline placebo	Infants GA 23 to 30 wks., BW 501–1,250 g, with respiratory failure requiring MV	45	23	22	NA	Neurodevelopmental outcome at 2 years CA	Early low-dose hydrocortisone showed no detectable adverse effect on neurologic status, growth, or respiratory history at 2 years.
Peltoniemi et al. (2016) (31)	5–7-year follow-up of Peltoniemi (2005) (29)	Finland	August 2002 to Mar 2006	5–7 years corrected age	Before 36 h of age	IV hydrocortisone 2.0 mg/kg/day for 2 days, then 1.5 mg/kg/day for 2 days, then 0.75 mg/kg/day for 6 days	Isotonic saline placebo	Very preterm infants (GA 23–30 weeks, BW 501–1,250 g) requiring MV within the first 24 h	37	18	19	NA	Neurodevelopmental, cognitive, speech, and growth outcomes at preschool age	Early neonatal hydrocortisone may be associated with adverse neurodevelopmental effects at 5–7 years
Shah et al. (2024) (33)	Single-center retrospective cohort study	USA	January 2017 to December 2021	Until 36 weeks PMA or hospital discharge	Within first 24 h after birth	Hydrocortisone 0.5 mg/kg twice daily for 7 days, then 0.5 mg/kg once daily for 3 days	No hydrocortisone	Extremely preterm infants born at 22–27 weeks' gestation admitted to NICU	287	82	205	Jensen criteria at 36 weeks PMA or discharge (Grade 0–3 based on respiratory support intensity)	Survival without BPD at 36 weeks PMA	Early prophylactic low-dose hydrocortisone was associated with improved adjusted survival without BPD and lower adjusted BPD rates
Smedbäck et al. (2026) (34)	National cohort study	Sweden	January 2018 and December 2023	36 wks. PMA	From day 1 of life	Hydrocortisone 1 mg/kg/day for days 1–7, then 0.5 mg/kg/day for days 8–10	No hydrocortisone	Extremely preterm infants born at 22–27 wks. GA admitted on day 1 of life at Swedish centers	1106	474	632	Need for supplemental oxygen at 36 wks. PMA	Survival without BPD at 36 wks. PMA	Prophylactic hydrocortisone was associated with improved adjusted survival without BPD and lower adjusted BPD incidence
Watterberg et al. (1999) (35)	Randomized, double-masked, placebo-controlled pilot trial	USA (2 centers)	June 1996 to May 1998	Until 36 weeks postconceptional age and hospital discharge	Before 48 h of life	Hydrocortisone 1 mg/kg/day for 9 days, then 0.5 mg/kg/day for 3 days	Normal saline placebo	Mechanically ventilated ELBW infants (BW 500–999 g), enrolled within 48 h of birth	40	20	20	Oxygen dependence at 36 weeks postconceptional age (CLD/BPD)	Survival without CLD/BPD at 36 weeks postconceptional age	Early low-dose hydrocortisone improved survival without CLD/BPD and reduced respiratory support duration
Watterberg et al. (2004) (36)	Multicenter randomized, masked, placebo-controlled trial	USA	November 2001 to April 2003	Until 36 weeks PMA and hospital discharge	Between 12 and 48 h of life	Hydrocortisone 1 mg/kg/day divided twice daily for 12 days, then 0.5 mg/kg/day for 3 days	Normal saline placebo	Mechanically ventilated ELBW infants (BW 500–999 g), enrolled within 12–48 h of life	360	180	180	Oxygen dependence at 36 weeks PMA	Survival without BPD at 36 weeks PMA	Early prophylactic hydrocortisone did not improve survival without BPD in the overall cohort, but improved survival without BPD and reduced mortality in infants exposed to histologic chorioamnionitis
Watterberg et al. (2007) (37)	18–22-month follow-up of Watterberg et al. (2004) (36)	USA	Original trial: Nov 2001 to Apr 2003	18–22 months	Between 12 and 48 h of life	Hydrocortisone 1 mg/kg/day divided twice daily for 12 days, then 0.5 mg/kg/day for 3 days	Normal saline placebo	Surviving ELBW infants from original trial; BW 500–999 g, mechanically ventilated at study entry	252	126	126	NA	Neurodevelopmental impairment and cerebral palsy at 18–22 months corrected age	Early low-dose hydrocortisone was not associated with increased cerebral palsy or neurodevelopmental impairment
Yao et al. (2025) (16)	Retrospective case-control study with propensity score matching	China	2020 to 2023	Until 36 weeks PMA/discharge	Within 24 h after birth	Hydrocortisone regimen: 1 mg/kg/day for 3 days, then 0.75 mg/kg/day for 2 days, followed by gradual tapering over the next 4 days	No early hydrocortisone	Extreme pre-term infants born at <26 weeks GA	66	33	33	BPD graded by respiratory support level at 36 weeks PMA	Incidence of Grade II + BPD	Early low-dose hydrocortisone was associated with reduced Grade II + BPD and shorter non-invasive ventilation duration
Purcell et al. (2026) (32)	Retrospective matched case-control study	United Kingdom	Oct 2017 to Dec 2021	Until 36 weeks	Within 24 h after birth	Early low-dose hydrocortisone (PREMILOC protocol; total 8.5 mg/kg over 10 days)	No early hydrocortisone	Extreme preterm neonates (<28 weeks GA)	188	94	94	Oxygen or respiratory support at 36 weeks CGA for ≥3 days	Survival without BPD at 36 weeks CGA	No improvement in survival without BPD; increased late-onset sepsis and longer respiratory support and hospital stay

GA, gestational age; BW, birth weight; NICU, neonatal intensive care unit; MV, mechanical ventilation; PMA, postmenstrual age; CA, corrected age; BPD, ronchopulmonary Dysplasia; CLD, chronic lung disease; PDA, patent ductus arteriosus; IVH, intraventricular hemorrhage; SIP, spontaneous intestinal perforation; NEC, necrotizing enterocolitis; PSM, propensity score matching; NDI, neurodevelopmental impairment; CP, cerebral palsy.

### 2.3 Outcomes

The primary outcomes were BPD and all-cause mortality before hospital discharge. The review did not impose a single definition of BPD. The definition used by each study was accepted, and these definitions are listed in Table 1. Some studies reported more than one definition. In these cases, the primary analysis used the definition based on oxygen requirement or respiratory support at 36 weeks' postmenstrual age. This definition was chosen because it was the one most often available across the included studies. If mortality before discharge was not reported, the nearest mortality time point was extracted for sensitivity analysis.

The secondary outcomes were survival without BPD, duration of respiratory support, use of rescue corticosteroids, PDA treatment, gastrointestinal and infectious complications, major neonatal morbidities, metabolic or cardiovascular adverse events, length of hospital stay, and neurodevelopmental impairment at follow-up.

### 2.4 Information sources and search strategy

A comprehensive literature search was performed in PubMed, the Cochrane Central Register of Controlled Trials, Scopus, and Web of Science. Each database was searched from inception to May 2026. The reference lists of included studies and relevant reviews were also screened by hand. The search strategy combined controlled vocabulary and free-text terms for prematurity, hydrocortisone, and BPD. No limit was placed on publication year. Non-English studies were eligible when sufficient data could be extracted. The full search strategy for each database is provided in Supplementary Table S1.

### 2.5 Study selection

All retrieved records were imported into EndNote™ 2025 (Clarivate, Philadelphia, PA, USA), and duplicates were removed. Two reviewers independently screened titles and abstracts against the eligibility criteria. The same reviewers then independently assessed the full texts of potentially relevant articles. Disagreements were resolved by discussion. If a disagreement remained, a third reviewer made the final decision. Reasons for exclusion at the full-text stage were recorded.

### 2.6 Data extraction

Two reviewers independently extracted data using a standardized form. The extracted variables were study design, setting, recruitment period, sample size, baseline infant characteristics, eligibility criteria, respiratory status, timing of randomization, follow-up, hydrocortisone regimen, and placebo details. Relevant co-interventions were also recorded, particularly PDA treatment with indomethacin or ibuprofen. Dichotomous outcomes were extracted as events and totals. Continuous outcomes were extracted as each study reported them, either as means with standard deviations or as medians with interquartile ranges or ranges. The conversion of medians to means is described under Data Synthesis and Statistical Analysis. When a trial had more than one report, the most complete dataset was used.

### 2.7 Risk-of-bias assessment

Two reviewers independently assessed the risk of bias in randomized trials using the Cochrane Risk of Bias 2 (RoB 2) tool (22). Five domains were assessed: the randomization process, deviations from intended interventions, missing outcome data, outcome measurement, and selection of the reported result. Each trial was judged to have a low risk of bias, some concerns, or a high risk of bias. Observational studies were assessed using the Newcastle–Ottawa Scale. Disagreements were resolved by consensus or by consultation with a third reviewer.

### 2.8 Data synthesis and statistical analysis

Meta-analysis was performed when at least two clinically comparable studies reported the same outcome. Dichotomous outcomes were summarized as risk ratios (RRs) with 95% confidence intervals (CIs). Risk differences were also calculated for clinically important outcomes when appropriate. Continuous outcomes were summarized as mean differences (MDs) with 95% CIs when the same measurement scale was used. Standardized mean differences were used when different scales measured the same construct.

Some studies reported continuous outcomes as medians with interquartile ranges (IQRs) or ranges. For these studies, the mean and standard deviation were estimated using the methods of Wan et al. and Luo et al. (23, 24), as recommended in the Cochrane Handbook (21). One study reported only the median and the width of the IQR. For this study, the median was used as the mean, and the standard deviation was estimated as the IQR width divided by 1.35 (21). These conversions were needed for only two outcomes: duration of mechanical ventilation and duration of oxygen therapy. The originally published medians are reported together with the pooled mean differences in the Results.

A random-effects model was used for all analyses because clinical heterogeneity was expected across studies. The main expected sources were gestational age, hydrocortisone dosing schedule, baseline respiratory risk, and neonatal co-interventions. Statistical heterogeneity was assessed with the chi-square test and quantified with the I^2^ statistic. I^2^ values of approximately 25%, 50%, and 75% were interpreted as low, moderate, and substantial heterogeneity, respectively. A continuity correction was applied when a study had zero events in one arm. Studies with zero events in both arms were excluded from the pooled risk ratio and were summarized narratively. All analyses were performed using Stata BE 18.5.

Subgroup analyses were performed only when at least two studies contributed data to each stratum. The prespecified subgroups were gestational age category, timing of hydrocortisone initiation, cumulative hydrocortisone dose, duration of treatment, baseline respiratory support, exposure to antenatal corticosteroids, and exposure to indomethacin or ibuprofen. Subgroup analyses by study design, chorioamnionitis exposure, and infant sex were added where studies reported stratified data. The included studies did not use a single definition of chorioamnionitis. Some used histological examination of the placenta, and others used a clinical diagnosis made by the treating team. None applied the criteria for intrauterine inflammation or infection or both (Triple I), which were proposed after most of these studies had been completed (25). The definition used by each study was therefore retained. Subgroups that did not meet the two-study threshold were not analyzed, and these are listed in the Results. Differences between subgroups were assessed with the test for subgroup differences.

A leave-one-out analysis was performed to test whether the pooled estimate depended on any single study. Publication bias and small-study effects were assessed with funnel plots when at least 10 studies were available for an outcome.

## 3 Results

### 3.1 Literature search and selection process

The database search retrieved 4,299 records: 681 from PubMed, 332 from Cochrane, 2,422 from Scopus, and 864 from Web of Science. After the removal of 591 duplicates, 3,708 records were screened by title and abstract, and 3,673 were excluded. The remaining 35 reports were all retrieved and assessed for eligibility. Of these, 21 were excluded: 2 were abstracts, 4 were reviews, and 15 did not meet the predefined PICO criteria. Finally, 14 studies with 3,416 infants were included in the systematic review and meta-analysis (16, 17, 26–37) (Figure 1).

**Figure 1:**
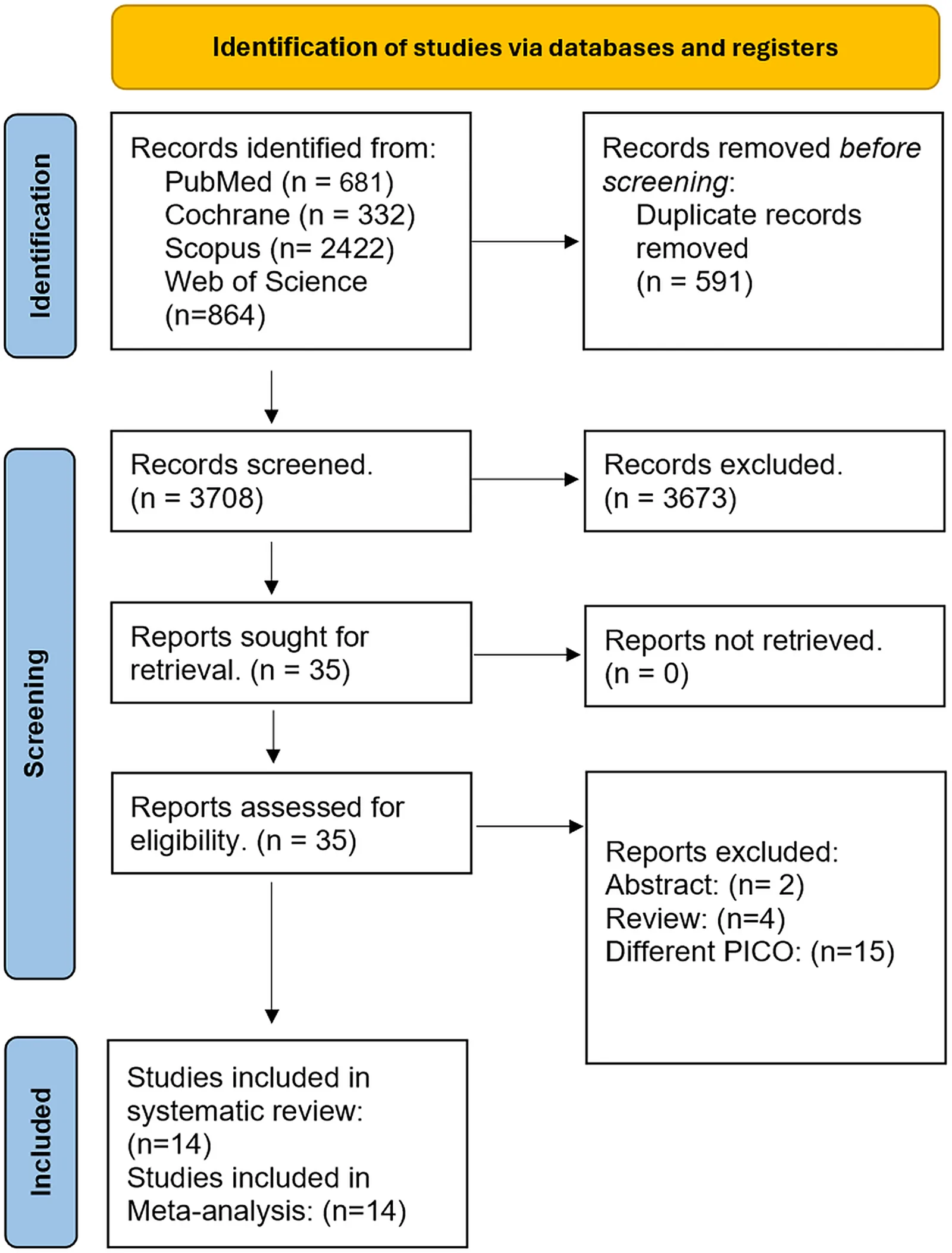
PRISMA flow diagram of study identification, screening, eligibility assessment, and inclusion.

### 3.2 Characteristics of the included studies

The 14 included studies comprised randomized placebo-controlled trials, follow-up reports of these trials, and observational cohort or case-control studies. The studies were conducted in France, Italy, Finland, the United States, Sweden, China, and the United Kingdom. Sample sizes ranged from 34 to 1,106 infants. Eligibility criteria for each study are summarized in the Methods and detailed in Table 1. Hydrocortisone was started between 3 and 48 h after birth. Most studies used low-dose regimens lasting 5–15 days. Follow-up most often extended to 36 weeks' postmenstrual age or hospital discharge. Several studies also reported neurodevelopmental outcomes at 18–22 months, 2 years, or 5–7 years. Mean gestational age at baseline ranged from 24.8 to 27.2 weeks, and mean birth weight ranged from approximately 698 to 958 g across study groups. Full study characteristics are presented in Tables 1, 2.

**Table 2:** Baseline characteristics of the included studies.

Author (year)	Group	Sample size (*N*)	Baseline infant characteristics						Baseline Maternal characteristics, *n* (%)				Apgar scores, mean (SD)	
			GA (weeks), mean (SD)	Birth weight (g), mean (SD)	Male sex, *n* (%)	Multiple birth, *n* (%)	Mechanical ventilation	Surfactant use	Antenatal Steroid	Cesarean delivery	Vaginal delivery	Chorioamnionitis	At 1 min	At 5 min
Baud et al. (2016) (17)	Hydrocortisone	255	26.4 (1.1)	860.0 (163.0)	131 (51)	82 (32)	NA	NA	238 (93)	79 (31)	131 (51)	114 (50)	NA	NA
	Placebo	266	26.4 (1.0)	848.3 (174.1)	149 (56)	90 (34)	NA	NA	246 (92)	70 (26)	143 (54)	127 (53)	NA	NA
Baud et al. (2017) (26)	Hydrocortisone	194	26.6 (0.9)	882 (149)	99 (51)	63 (32)	NA	NA	187 (96)	NA	101 (52)	88 (49)	NA	NA
	Placebo	185	26.5 (0.9)	888 (156)	106 (57)	64 (35)	NA	NA	177 (96)	NA	97 (52)	91 (53)	NA	NA
Bonsante et al. (2007) (27)	Hydrocortisone	25	26.3 (0.55)	845 (75)	13 (52)	NA	25 (100)	25 (100)	13 (52)	NA	NA	9 (36)	NA	NA
	Placebo	25	26.4 (0.78)	885 (70)	16 (64)	NA	25 (100)	25 (100)	17 (68)	NA	NA	13 (52)	NA	NA
Efird et al. (2005) (28)	Hydrocortisone	16	26 (1)	775 (183)	6 (38)	11 (69)	NA	NA	16 (100)	13 (81)	3 (19)	NA	6.33 (1.50)	7 (1.25)
	Placebo	18	26 (1)	790 (142)	9 (50)	9 (50)	NA	NA	17 (94)	13 (72)	5 (28)	NA	5.00 (2.00)	6 (1.75)
Peltoniemi et al. (2005) (29)	Hydrocortisone	25	26.7 (1.6)	888 (204)	16 (64)	5 (20)	25 (100)	24 (96)	23 (92)	11 (44)	14 (56)	9 (36)	4 (1.11)	7.00 (1.85)
	Placebo	26	26.5 (2.8)	903 (220)	14 (54)	7 (27)	26 (100)	23 (89)	25 (96)	13 (50)	13 (50)	9 (35)	4.50 (3.33)	6.50 (4.44)
Peltoniemi et al. (2009) (30)	Hydrocortisone	23	26.8 (1.5)	903 (206)	14 (61)	NA	23 (100)	22 (96)	21 (91)	NA	NA	NA	4.0 (1.85)	NA
	Placebo	22	27.1 (1.5)	949 (207)	12 (55)	NA	22 (100)	19 (86)	21 (96)	NA	NA	NA	4.5 (1.11)	NA
Peltoniemi et al. (2016) (31)	Hydrocortisone	18	26.8 (1.6)	875 (202)	9 (50)	NA	18 (100)	17 (94)	NA	NA	NA	NA	4.5 (1.75)	5.5 (1.75)
	Placebo	19	27.2 (1.5)	958 (198)	11 (58)	NA	19 (100)	17 (90)	NA	NA	NA	NA	4.0 (1.50)	5.5 (1.25)
Shah et al. (2024) (33)	Hydrocortisone	82	25.0 (1.48)	720.0 (170.4)	39 (47.6)	3 (3.7)	NA	72 (87.8)	62 (75.6)	42 (51.2)	NA	2 (2.4)	NA	NA
	No hydrocortisone	205	25.7 (2.22)	790.0 (192.6)	106 (51.7)	20 (9.7)	NA	180 (87.9)	150 (73.8)	139 (68.4)	NA	3 (1.5)	NA	NA
Smedbäck et al. (2026) (34)	Hydrocortisone	474	25.4 (2.07)	769.3 (257.0)	257 (54.2)	99 (20.9)	NA	365 (77.0)	425 (94.0)	NA	NA	NA	NA	NA
	Control	632	25.9 (1.93)	792.7 (265.2)	346 (54.7)	159 (25.2)	NA	470 (74.4)	536 (91.0)	NA	NA	NA	NA	NA
Watterberg et al. (1999) (35)	Hydrocortisone	20	25.2 (1.3)	732 (135)	6 (30)	NA	20 (100)	NA	17 (85)	NA	NA	14 (70)	NA	NA
	Placebo	20	25.4 (1.5)	770 (135)	9 (45)	NA	20 (100)	NA	17 (85)	NA	NA	8 (42)	NA	NA
Watterberg et al. (2004) (36)	Hydrocortisone	180	25.2 (1.5)	731 (126)	96 (53)	42 (23)	180 (100)	152 (84)	138 (77)	NA	77 (43)	73 (53)	4.0 (2.96)	7.0 (1.48)
	Placebo	180	25.3 (1.7)	734 (126)	90 (50)	38 (21)	180 (100)	158 (88)	146 (81)	NA	63 (35)	76 (52)	4.0 (2.96)	6.7 (2.22)
Watterberg et al. (2007) (37)	Hydrocortisone	126	25.3 (1.5)	738 (122)	68 (54)	NA	126 (100)	NA	NA	NA	NA	57/103 (55)	NA	NA
	Placebo	126	25.4 (1.6)	738 (124)	64 (51)	NA	126 (100)	NA	NA	NA	NA	55/107 (51)	NA	NA
Yao et al. (2025) (16)	Hydrocortisone	33	24.78 (0.85)	698 (102)	20 (60.6)	NA	NA	32 (96.9)	24 (72.7)	10 (30.3)	NA	12 (36.4)	5.0 (2.96)	8.0 (2.22)
	No hydrocortisone	33	24.75 (0.80)	747 (101)	22 (66.7)	NA	NA	21 (63.6)	22 (66.7)	5 (15.2)	NA	11 (33.0)	5.0 (2.22)	8.0 (1.48)
Purcell et al. (2026) (32)	Hydrocortisone	94	25.45 (1.28)	821 (182)	56 (59.5)	21 (22.3)	NA	NA	66 (70.2)	NA	NA	10 (10.6)	NA	NA
	No hydrocortisone	94	25.46 (1.25)	825.4 (178)	56 (59.5)	21 (22.3)	NA	NA	75 (79.8)	NA	NA	13 (13.8)	NA	NA

### 3.3 Risk of bias

Risk of bias was assessed at the trial level with the RoB 2 tool for the six randomized trials included in the primary analysis. Four trials had a low overall risk of bias. Two trials had some concerns, mainly in the randomization process and in the selection of the reported result. The risk of bias from deviations from intended interventions, missing outcome data, and outcome measurement was generally low (Figure 2).

**Figure 2:**
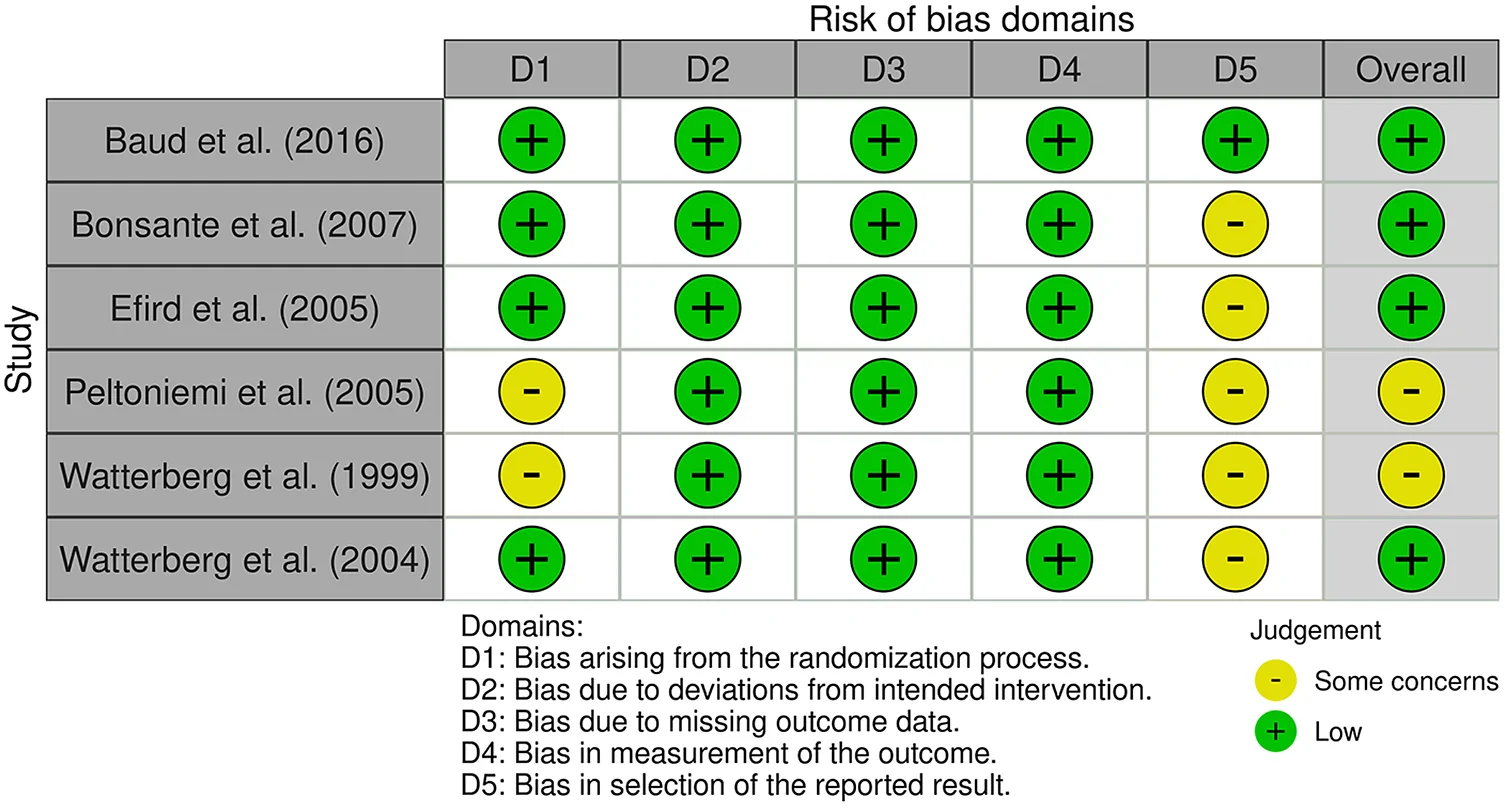
Risk-of-bias assessment of randomized controlled trials using the cochrane roB 2 tool.

Four additional reports were follow-up publications of these randomized trials. They did not represent independent study populations or separate randomization processes. A separate risk-of-bias assessment was therefore not performed for these reports, and the judgment for the parent trial was applied.

Observational studies were assessed with the Newcastle–Ottawa Scale. Both case-control studies were rated as high quality, with total scores of 9. The two cohort studies were also rated as high quality, with total scores of 8 and 9 (Supplementary Tables S2, S3).

### 3.4 Primary outcomes

Early hydrocortisone was not associated with a significant reduction in BPD incidence at 36 weeks' PMA (RR: 0.94, 95% CI: 0.85–1.04, *p* = 0.25) (Figure 3).

**Figure 3:**
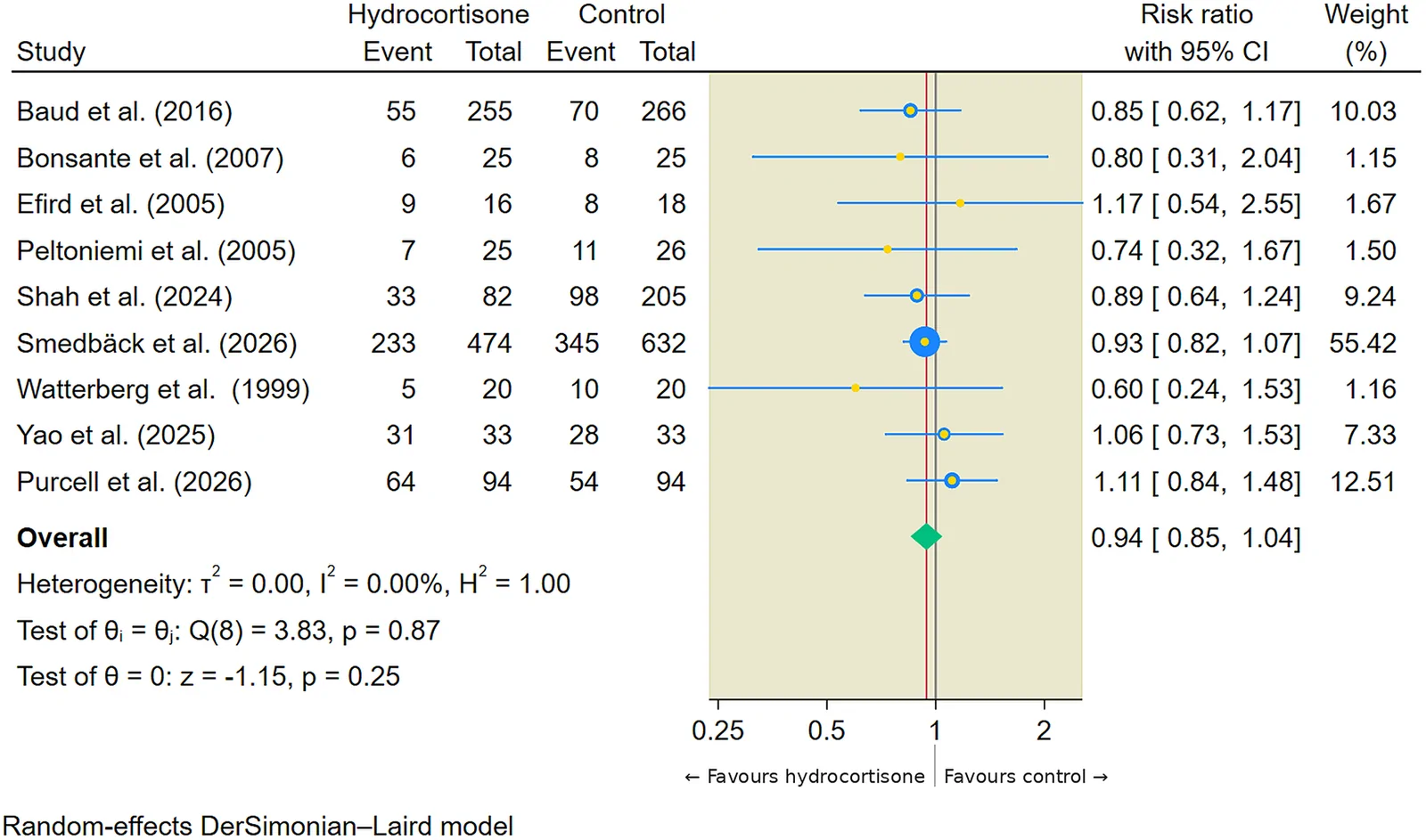
Effect of early prophylactic hydrocortisone on bronchopulmonary dysplasia at 36 weeks' postmenstrual age. Estimates to the left of the line of no effect favour hydrocortisone; estimates to the right favour control.

For all-cause mortality before hospital discharge, no significant difference was observed between groups (RR: 1.07, 95% CI: 0.77–1.49, *p* = 0.69) (Figure 4). Death before 36 weeks' postmenstrual age was likewise comparable between groups (RR: 1.00, 95% CI: 0.84–1.20, *p* = 0.99) (Figure 5).

**Figure 4:**
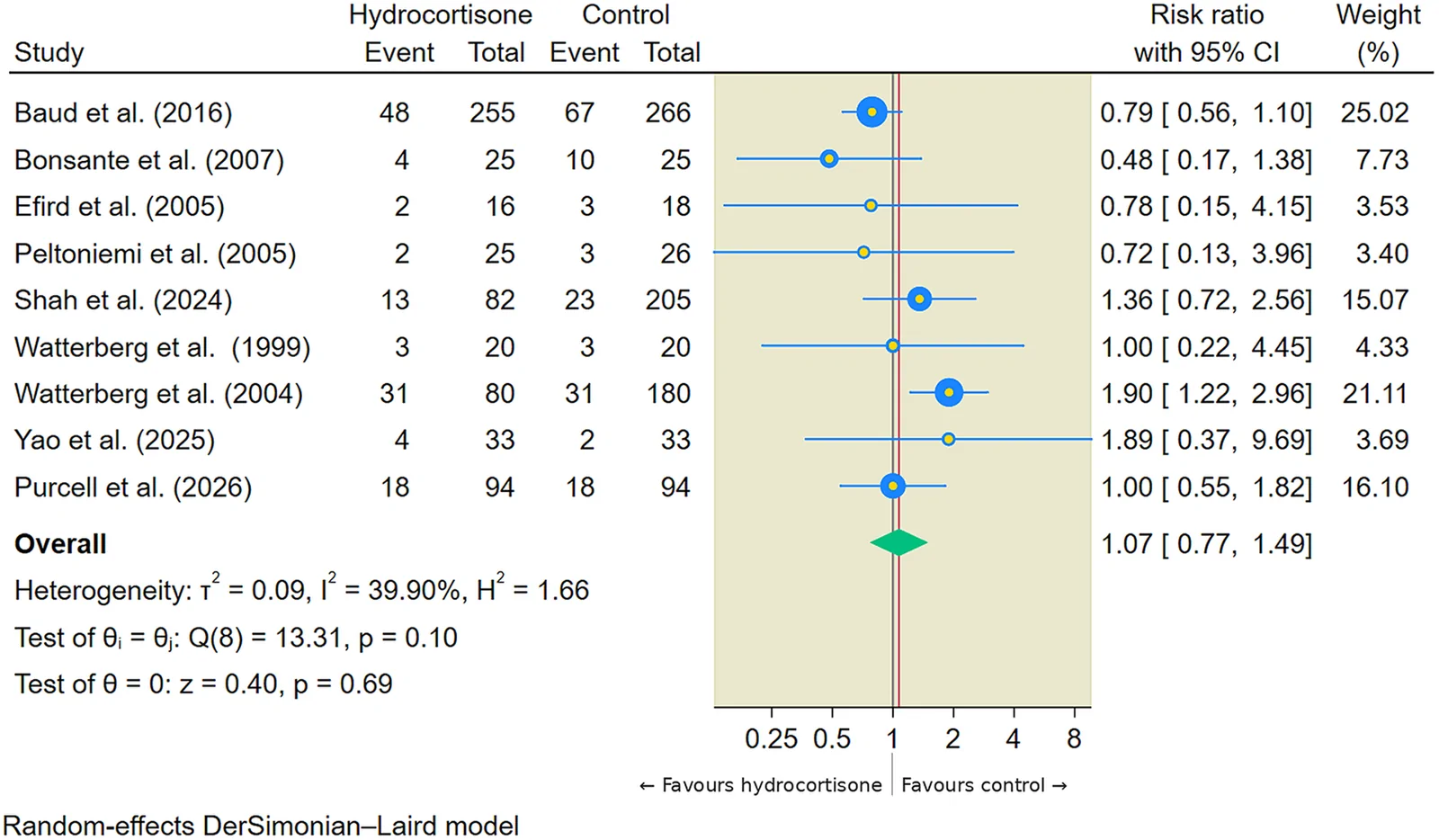
Effect of early prophylactic hydrocortisone on all-cause mortality before hospital discharge. Estimates to the left of the line of no effect favour hydrocortisone; estimates to the right favour control.

**Figure 5:**
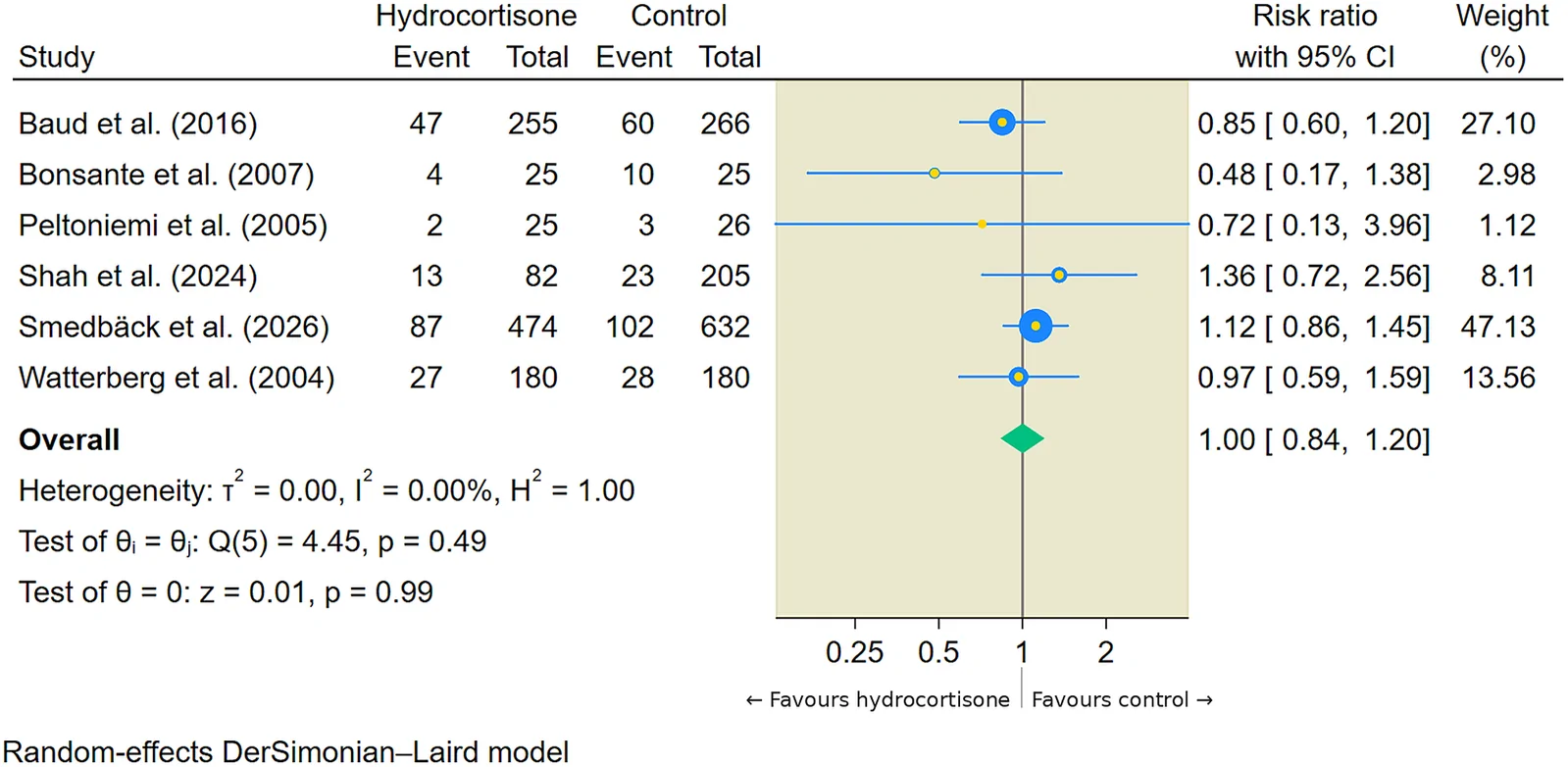
Effect of early prophylactic hydrocortisone on death before 36 weeks' postmenstrual age. Estimates to the left of the line of no effect favour hydrocortisone; estimates to the right favour control.

### 3.5 Secondary outcomes

#### 3.5.1 Survival without bronchopulmonary dysplasia at 36 weeks' postmenstrual age

Ten studies with 2,703 infants reported this outcome. Survival without BPD occurred in 471 of 1,204 infants in the hydrocortisone group and in 529 of 1,499 infants in the control group. Early hydrocortisone was not associated with a statistically significant improvement in survival without BPD (RR: 1.07, 95% CI: 0.97–1.19, *p* = 0.18). No meaningful heterogeneity was observed (I^2^ = 0%, *p* = 0.76) (Figure 6).

**Figure 6:**
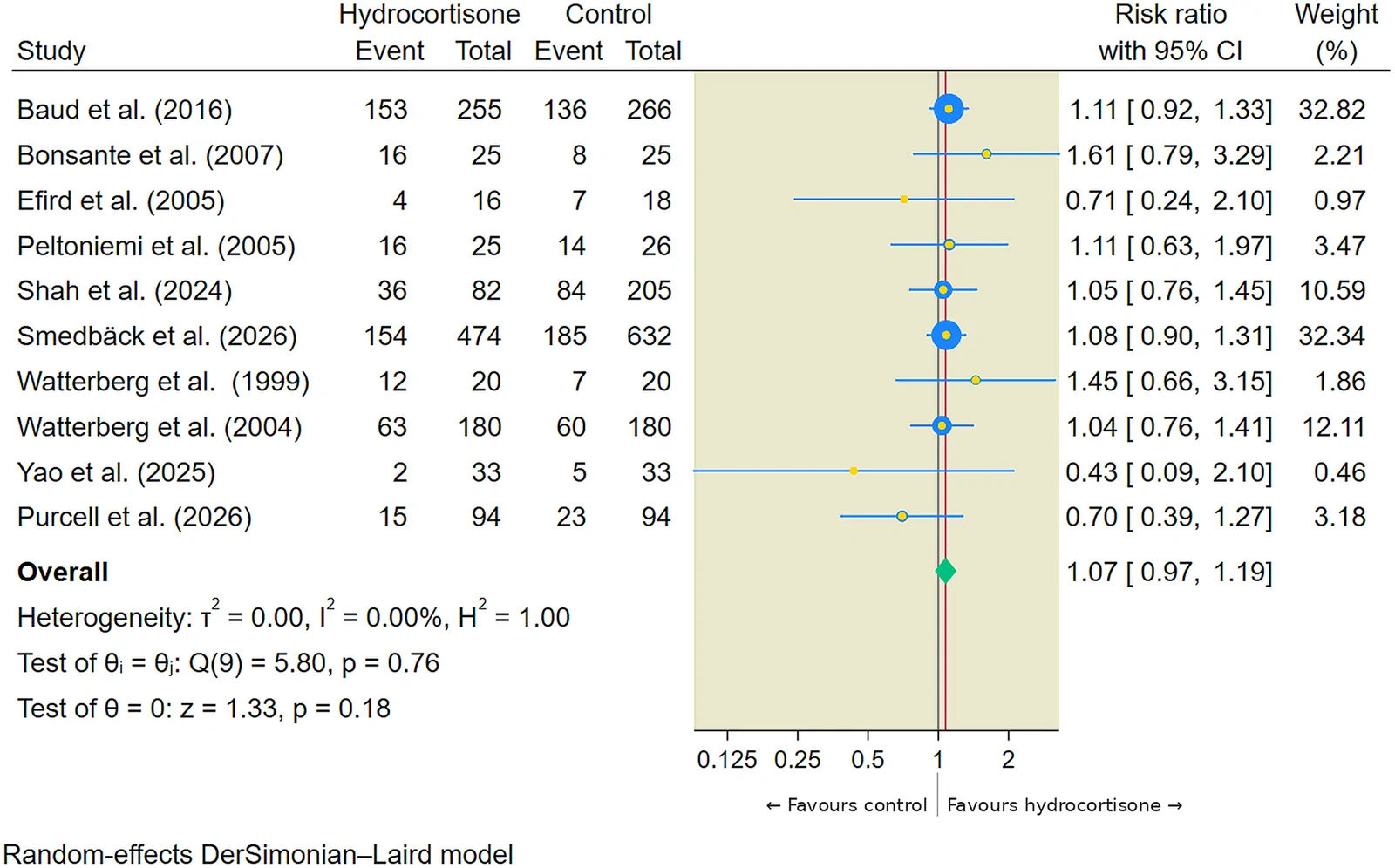
Effect of early prophylactic hydrocortisone on survival without bronchopulmonary dysplasia at 36 weeks' postmenstrual age. Estimates to the right of the line of no effect favour hydrocortisone; estimates to the left favour control.

Subgroup analyses showed no statistically significant modification of the treatment effect across the examined strata. According to timing of hydrocortisone initiation, studies initiating treatment within 24 h showed no significant association with survival without BPD (RR: 1.06, 95% CI: 0.94–1.19, *p* = 0.34; I^2^ = 0%, *p* = 0.57), and studies initiating treatment within 48 h showed a similar non-significant estimate (RR: 1.14, 95% CI: 0.90–1.45, *p* = 0.29; I^2^ = 0%, *p* = 0.65). The test for subgroup differences was not statistically significant (*p* = 0.59) (Figure 7).

**Figure 7:**
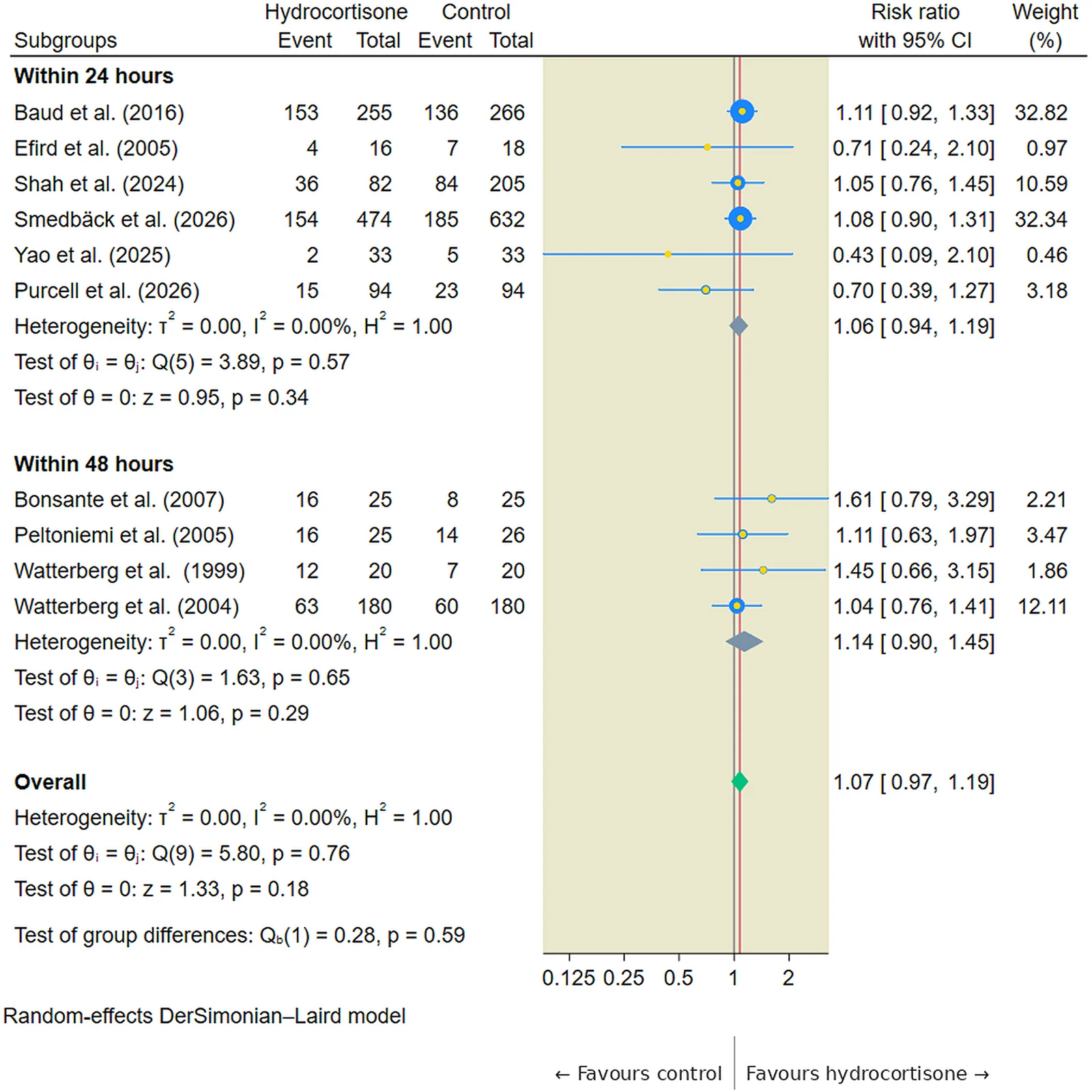
Subgroup analysis of survival without bronchopulmonary dysplasia according to timing of hydrocortisone initiation. Estimates to the right of the line of no effect favour hydrocortisone; estimates to the left favour control.

By study design, the pooled estimate across RCTs revealed no statistically significant difference (RR: 1.11, 95% CI: 0.96–1.28, *p* = 0.16; I^2^ = 0%, *p* = 0.80). Observational studies also showed no significant association (RR: 1.03, 95% CI: 0.88–1.21, *p* = 0.70), with minimal heterogeneity (I^2^ = 1.8%, *p* = 0.38). The subgroup difference between randomized and observational evidence was not significant (*p* = 0.51) (Figure 8).

**Figure 8:**
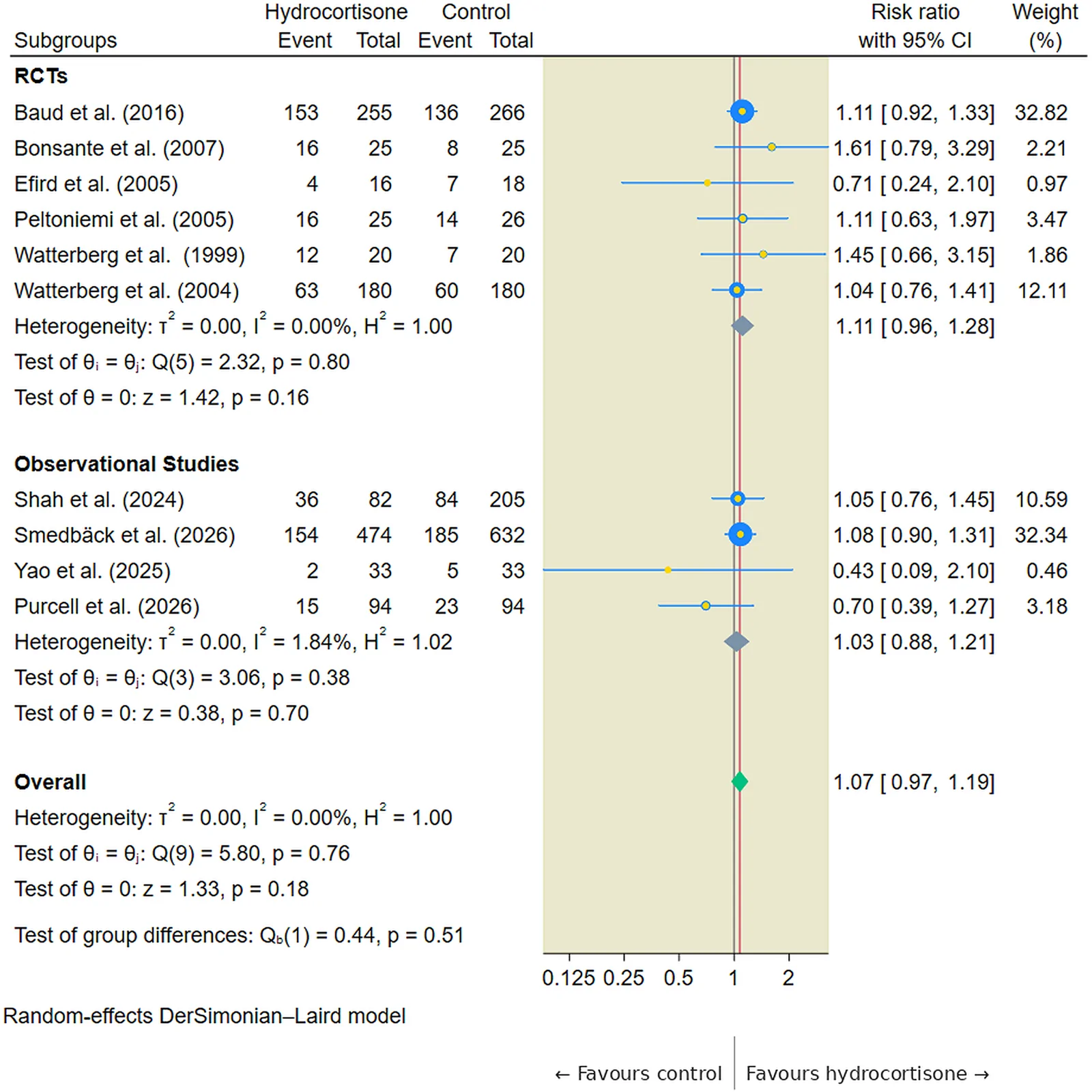
Subgroup analysis of survival without bronchopulmonary dysplasia according to study design. Estimates to the right of the line of no effect favour hydrocortisone; estimates to the left favour control.

In the chorioamnionitis subgroup, the effect estimate favored hydrocortisone among infants exposed to chorioamnionitis, with higher survival without BPD compared with control (RR: 1.26, 95% CI: 1.01–1.56). Among infants without chorioamnionitis, the estimate was smaller and non-significant (RR: 1.08, 95% CI: 0.93–1.25) (Figure 9).

**Figure 9:**
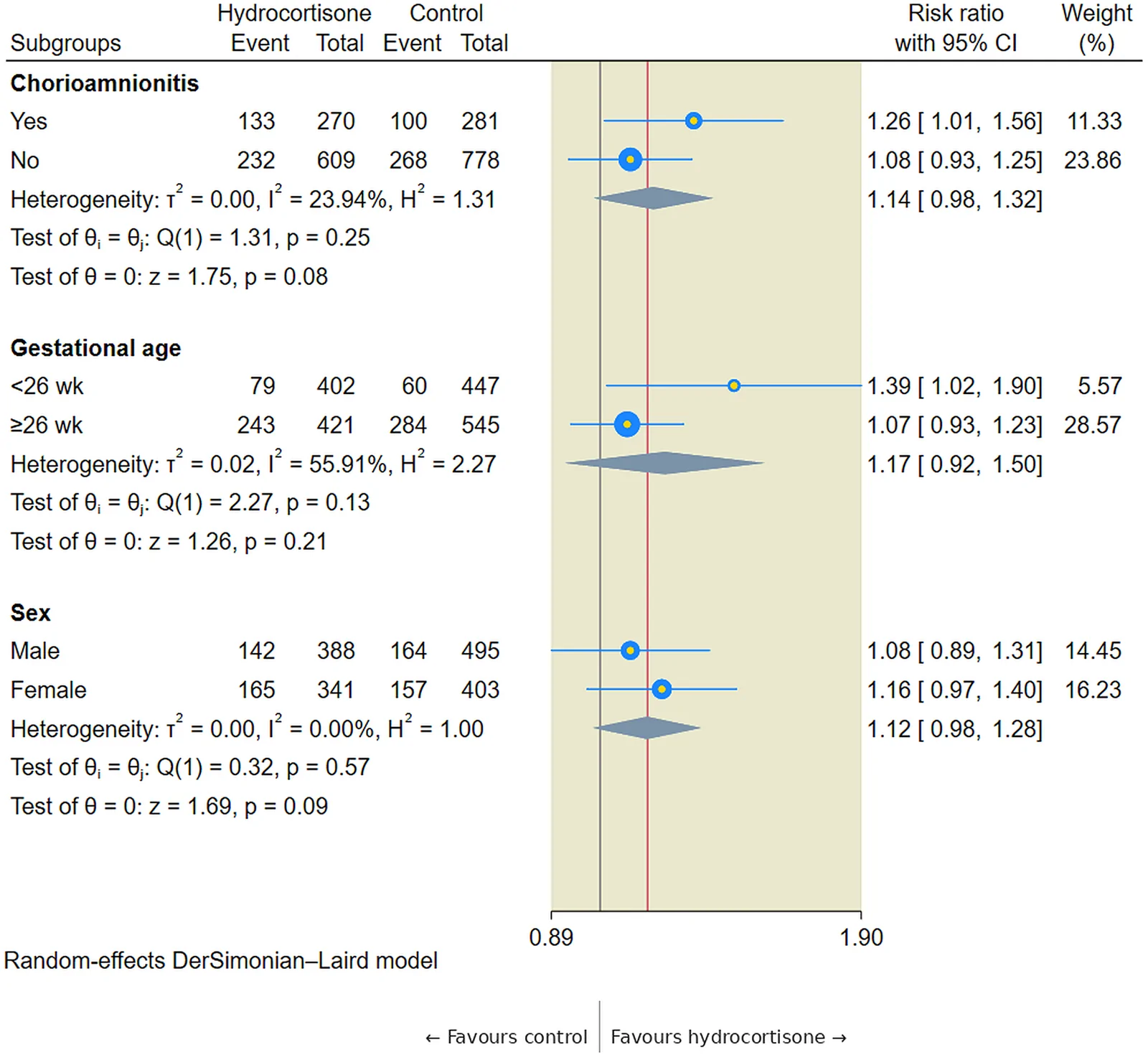
Subgroup analysis of survival without bronchopulmonary dysplasia according to chorioamnionitis exposure, gestational age, and sex. Estimates to the right of the line of no effect favour hydrocortisone; estimates to the left favour control.

Among infants born before 26 weeks, survival without BPD was significantly higher with hydrocortisone (RR: 1.39, 95% CI: 1.02–1.90). Among infants born at 26 weeks or later, the estimate was smaller and crossed the null (RR: 1.07, 95% CI: 0.93–1.23) (Figure 9).

By sex, the effect estimates were broadly similar and non-significant between males and females. Among males, the RR was 1.08, 95% CI: 0.89–1.31, while among females, the RR was 1.16, 95% CI: 0.97–1.40 **(**Figure 9).

Subgroup analyses for cumulative hydrocortisone dose, duration of treatment, baseline respiratory support, exposure to antenatal corticosteroids, and exposure to indomethacin or ibuprofen were prespecified but could not be performed. No included study reported outcome data stratified by these variables. The prespecified threshold of at least two studies per stratum was therefore not met.

In the leave-one-out sensitivity analysis, the exclusion of individual studies did not materially change the pooled estimate. The risk ratios ranged from 1.06 to 1.09, and all confidence intervals crossed the null (Supplementary Figure S1). Visual inspection of the funnel plot suggested mild asymmetry around the pooled estimate, which may indicate publication bias (Supplementary Figure S2).

#### 3.5.2 Respiratory support outcomes

Early hydrocortisone was not associated with a significant difference in the duration of mechanical ventilation (MD: −1.11 days, 95% CI: −4.67–2.46, *p* = 0.54) or the duration of oxygen therapy (MD: −5.81 days, 95% CI: −14.55–2.93, *p* = 0.19). Several studies reported these outcomes as medians. Their originally published values are given below for the hydrocortisone and control groups, respectively. For duration of mechanical ventilation, Bonsante et al. (27) reported a median of 4 days (range 2–21) versus 15 days (range 2–27). Peltoniemi et al. (29) reported 4 days (IQR 2–20) versus 13 days (IQR 2–40). Watterberg et al. (35) reported 25 days (IQR 14–34) versus 32 days (IQR 11–45) among survivors. Watterberg et al. (36) reported 32 days (IQR 13–54) versus 35 days (IQR 17–47). Yao et al. (16) reported 22 days in both groups, with IQR widths of 27 and 29 days. The other two studies reported means: Efird et al. (28) with standard deviations and Smedbäck et al. (34) with 95% confidence intervals. For duration of oxygen therapy, all four contributing studies reported medians. Peltoniemi et al. (29) reported 34 days (IQR 19–59) versus 62 days (IQR 30–110). Watterberg et al. (35) reported 48 days (IQR 32–64) versus 69 days (IQR 34–75) on more than 25% inspired oxygen. Watterberg et al. (36) reported 69 days (IQR 54–78) versus 69 days (IQR 56–81). Yao et al. (16) reported 90 days versus 91 days, with IQR widths of 42 and 44 days. Means and standard deviations were estimated from these values as described in the Methods. The need for oxygen at discharge was also similar between groups (RR: 0.98, 95% CI: 0.78–1.23, *p* = 0.84) (Supplementary Figures S3–S5).

#### 3.5.3 Neonatal safety and systemic complications

Early hydrocortisone was not associated with a significant difference in gastrointestinal perforation (RR: 1.34, 95% CI: 0.93–1.93, *p* = 0.12) or necrotizing enterocolitis (RR: 1.04, 95% CI: 0.82–1.32, *p* = 0.74) (Supplementary Figures S6, S7). However, late-onset sepsis was higher in the hydrocortisone group (RR: 1.19, 95% CI: 1.05–1.35, *p* = 0.01) (Supplementary Figure S8). No significant differences were observed for hypertension requiring treatment (RR: 1.11, 95% CI: 0.38–3.25, *p* = 0.85) (Supplementary Figure S9) or insulin requirement (RR: 1.07, 95% CI: 0.94–1.22, *p* = 0.32) (Supplementary Figure S10).

For PDA-related outcomes, early hydrocortisone was not significantly associated with medically treated PDA (RR: 0.79, 95% CI: 0.61–1.03, *p* = 0.08) or surgically treated PDA (RR: 0.83, 95% CI: 0.63–1.11, *p* = 0.21) **(**Supplementary Figure S1^1^). Similarly, no significant differences were found for severe intraventricular hemorrhage grade 3–4 (RR: 1.00, 95% CI: 0.82–1.22, *p* = 0.98) (Supplementary Figure S1^2^), periventricular leukomalacia (RR: 0.69, 95% CI: 0.37–1.28, *p* = 0.24) (Supplementary Figure S1^3^), retinopathy of prematurity at any stage (RR: 1.00, 95% CI: 0.88–1.13, *p* = 0.97), retinopathy of prematurity grade ≥ III (RR: 0.95, 95% CI: 0.74–1.22, *p* = 0.68) (Supplementary Figure S1^4^), or pulmonary hemorrhage (RR: 1.13, 95% CI: 0.80–1.62, *p* = 0.48) (Supplementary Figure S1^5^).

#### 3.5.4 Neurodevelopmental and sensory outcomes

Early hydrocortisone was not associated with a significant difference in neurodevelopmental impairment at 2-year follow-up (RR: 0.93, 95% CI: 0.74–1.17, *p* = 0.53) or 5–7-year follow-up (RR: 1.24, 95% CI: 0.54–2.81, *p* = 0.61) (Supplementary Figure S1^6^). Similarly, no significant differences were observed for cerebral palsy at 2 years (RR: 1.02, 95% CI: 0.63–1.68, *p* = 0.92) or 5–7 years (RR: 4.76, 95% CI: 0.24–93.19, *p* = 0.30) (Supplementary Figure S1^7^).

For cognitive outcomes, hydrocortisone was not associated with cognitive impairment at 2 years (RR: 0.80, 95% CI: 0.55–1.17, *p* = 0.25) or 5–7 years (RR: 2.11, 95% CI: 0.21–21.32, *p* = 0.53) (Supplementary Figure S1^8^). Auditory impairment was also not significantly different at 2 years (RR: 0.46, 95% CI: 0.13–1.61, *p* = 0.22) or 5–7 years (RR: 3.00, 95% CI: 0.13–69.09, *p* = 0.49) (Supplementary Figure S1^9^). Visual impairment showed no significant difference at 2 years (RR: 0.93, 95% CI: 0.56–1.53, *p* = 0.77) or 5–7 years (RR: 1.06, 95% CI: 0.02–50.43, *p* = 0.98) **(**Supplementary Figure S20).

## 4 Discussion

### 4.1 Summary of main findings

In this systematic review and meta-analysis of 14 studies including 3,416 infants, early prophylactic hydrocortisone did not significantly improve the main respiratory outcomes. Survival without BPD at 36 weeks' postmenstrual age was numerically higher with hydrocortisone but did not reach statistical significance, and there was no significant reduction in BPD at 36 weeks, death before 36 weeks, or all-cause mortality before discharge. Respiratory support outcomes, including duration of mechanical ventilation, duration of oxygen therapy, and oxygen requirement at discharge, were also similar between groups. Subgroup analyses suggested possible benefit among infants exposed to chorioamnionitis and those born before 26 weeks' gestation, but these findings should be considered exploratory. Early hydrocortisone was associated with an increased risk of late-onset sepsis, while gastrointestinal perforation, necrotizing enterocolitis, patent ductus arteriosus treatment, severe brain injury, retinopathy of prematurity, and measured neurodevelopmental outcomes did not differ significantly between groups.

### 4.2 Biological rationale and respiratory outcomes

The neutral effect of early prophylactic hydrocortisone on BPD-related outcomes should be interpreted within the complex biology of lung injury in extremely preterm infants. The benefit of hydrocortisone is biologically plausible because the first postnatal days involve inflammatory, respiratory, and hemodynamic instability, often with limited adrenal reserve and inadequate cortisol response (38–40). Supplementation may therefore support cardiovascular adaptation, reduce inflammatory lung injury, and promote respiratory stabilization. However, BPD is not driven by a single pathway. It reflects the combined effects of extreme lung immaturity, impaired alveolar and vascular development, antenatal inflammation, oxygen exposure, mechanical ventilation, infection, nutrition, and differences in neonatal care (3, 5, 41, 42). Thus, targeting inflammation and adrenal insufficiency alone may be insufficient to consistently reduce BPD. This may explain the absence of a significant overall improvement in survival without BPD or BPD at 36 weeks, despite some numerically favorable trends.

### 4.3 Clinical heterogeneity and subgroup findings

The included studies varied in several clinically important respects. Infants were enrolled across a gestational age range of approximately 22–30 weeks, and the baseline risk of BPD differs substantially across this range (3, 5, 41, 42). Hydrocortisone was initiated within the first 3–48 h after birth, but dose, duration, cumulative exposure, and co-interventions were not identical across studies. Respiratory management strategies, thresholds for oxygen use, patent ductus arteriosus treatment, infection prevention practices, and definitions of BPD may also have differed. These sources of heterogeneity can dilute a treatment effect if hydrocortisone benefits only a narrower subgroup of infants. The subgroup findings are therefore important. The apparent benefit among infants exposed to chorioamnionitis may reflect a subgroup in whom antenatal inflammation contributes more directly to early pulmonary injury.

In a systematic review and meta-analysis of about a quarter of a million preterm infants, chorioamnionitis was significantly associated with BPD. The odds ratio was 2.32 for BPD defined at 28 days and 1.29 for BPD defined at 36 weeks' postmenstrual age (43). Hydrocortisone may be more effective when inflammation is already a major driver of disease. A meta-analysis by Zhou et al. found that among infants weighing less than 1,000 g, those exposed to chorioamnionitis may represent a subgroup in which early low-dose hydrocortisone confers protection against BPD or death (44). Similarly, infants born before 26 weeks' gestation have the greatest developmental immaturity and the highest baseline risk of BPD or death (45). A therapy with modest absolute benefit may be easier to detect in such a high-risk group. However, these subgroup effects require confirmation in adequately powered analyses, preferably using individual patient data, because study-level subgroup findings may be influenced by differences in case mix, trial conduct, and co-interventions.

### 4.4 Definition of chorioamnionitis

The chorioamnionitis subgroup finding should be interpreted with caution, because chorioamnionitis was not diagnosed in the same way in every study. There is no universally accepted clinical definition. In practice, the diagnosis is made in one of two ways. The first is histological examination of the placenta. This method is comparatively specific, but it is not performed in every case. The second is a clinical diagnosis based on maternal fever together with signs such as maternal or fetal tachycardia, uterine tenderness, or purulent amniotic fluid. This approach is sensitive but non-specific, and it overlaps with non-infectious causes of fever during labor. For this reason, a National Institute of Child Health and Human Development expert panel proposed replacing the term with “intrauterine inflammation or infection or both” (Triple I) and applying explicit diagnostic criteria (25). In the studies reviewed here, chorioamnionitis was diagnosed histologically in some and clinically in others. Not every study reported its definition in full. No study applied the Triple I criteria, and most had completed recruitment before these criteria were proposed. The chorioamnionitis subgroup therefore combines several definitions. For this reason, the subgroup estimate should be regarded as hypothesis-generating and should not be used to select infants for treatment.

### 4.5 Mortality

Early hydrocortisone did not significantly reduce death before 36 weeks' postmenstrual age or all-cause mortality before discharge, suggesting that any possible respiratory benefit was not accompanied by a measurable survival advantage in the overall pooled population. At the same time, the findings do not suggest an early mortality penalty, although the confidence interval for mortality before discharge remained wide. Mortality in extremely preterm infants is driven by several competing pathways, including respiratory failure, sepsis, necrotizing enterocolitis, severe brain injury, and decisions regarding continuation of intensive care (46, 47). Therefore, a single early intervention is unlikely to alter survival unless its effect is large or concentrated in a clearly defined high-risk subgroup.

### 4.6 Safety outcomes

Safety outcomes remain central to the interpretation of these findings. The increased risk of late-onset sepsis is clinically important because hydrocortisone has immunomodulatory effects, and extremely preterm infants already have immature immune defenses, prolonged hospitalization, and frequent invasive device exposure (19, 48, 49). Gastrointestinal perforation was not significantly increased, but the point estimate was above unity, and risk may be concentrated in specific contexts such as concurrent indomethacin exposure, severe illness, or extreme immaturity. The absence of a significant increase in necrotizing enterocolitis and the lack of clear differences in neurodevelopmental, cognitive, visual, or auditory outcomes are reassuring. However, the longer-term estimates were imprecise. This imprecision reflects how little evidence is available. Outcomes at 5–7 years came from the follow-up of a single trial. Event counts were small, and the confidence intervals were therefore wide. Pooling the existing published studies again will not improve precision at these time points. Better precision requires follow-up of already-randomized cohorts to school age, harmonized outcome definitions, and pooling of individual participant data. Overall, these findings support careful monitoring for infection and gastrointestinal complications. Early hydrocortisone should not be regarded as a risk-free prophylactic strategy.

### 4.7 Comparison with previous evidence

Previous evidence syntheses have reached different conclusions. The main reason is that they addressed different clinical questions and combined different populations, corticosteroid agents, treatment windows, and analytical approaches. Earlier hydrocortisone-specific reviews suggested that systemic or prophylactic low-dose hydrocortisone may improve survival without BPD in preterm infants. These reviews also reported safety concerns, particularly late-onset sepsis and gastrointestinal perforation. The perforation signal was most evident when hydrocortisone was given together with indomethacin for PDA (14, 19). In contrast, broader network meta-analyses of corticosteroids, including those by Ramaswamy et al. (50) and Hay et al. (51), generally found that dexamethasone regimens had stronger effects on BPD-related outcomes. In these analyses, the benefit of hydrocortisone was more modest or less consistent, and uncertainty remained about uncommon adverse events and long-term neurodevelopment. More recent reviews have emphasized that the treatment effect may not be the same in all preterm infants. It may depend on baseline risk, gestational age, timing, treatment duration, and inflammatory exposure such as chorioamnionitis (52, 53).

The present review differs from much of the earlier literature in two ways. It focused only on early prophylactic hydrocortisone, and it included a larger evidence base of 14 studies and 3,416 infants from randomized trials, follow-up studies, and observational studies. Unlike some earlier hydrocortisone-focused analyses, our pooled results did not show a statistically significant improvement in survival without BPD. They also did not show a significant reduction in BPD at 36 weeks' postmenstrual age, death before 36 weeks, or all-cause mortality before discharge.

Several features of the evidence base may explain why our pooled estimates are more neutral than some earlier reports. The included studies span nearly three decades of neonatal practice, from trials that recruited in the late 1990s to cohorts that recruited in the 2020s. Neonatal care changed substantially over this period. Changes occurred in antenatal corticosteroid coverage, surfactant strategy, non-invasive respiratory support, oxygen saturation targets, nutrition, and infection control. The baseline risk of BPD and the survival of the most immature infants changed as well (2). The effect of a therapy that depends on baseline inflammatory and respiratory risk may therefore differ between earlier and later studies. Pooling across the whole period may weaken an effect that is present in only part of it. An analysis restricted to studies from the last 15 years would test this possibility directly. It would include fewer but more contemporary studies, mainly PREMILOC and the recent national and single-center cohorts. We did not perform this analysis because it was not prespecified in the protocol, and a *post hoc* analysis limited by date would carry a risk of selective reporting. We propose it as an objective for a future update of this review.

### 4.8 Inclusion of observational studies

The decision to include comparative observational studies also influenced the results. Including them approximately doubled the number of infants in the primary comparison. It also added contemporary real-world data from national and single-center cohorts, which no current randomized trial provides for this indication. The disadvantage is that observational studies cannot exclude confounding by indication. Infants who receive prophylactic hydrocortisone in routine care are not selected at random, and units that adopt the practice early may differ from other units in other respects. We addressed this limitation in three ways. We appraised the observational studies with the Newcastle–Ottawa Scale, we analyzed study design as a subgroup, and we reported the randomized and observational estimates separately. The two estimates were consistent with each other and with the overall pooled result. Readers who prefer to consider only randomized evidence can use the estimate stratified by study design. The main conclusion of this review does not depend on the observational studies.

### 4.9 Limitations

Several limitations should be acknowledged. The number of randomized trials was limited, and some outcomes were reported by only a few studies. This reduced the statistical power for uncommon adverse events and long-term outcomes. Clinical heterogeneity was present in gestational age, hydrocortisone regimen, timing of initiation, PDA treatment, respiratory care, and BPD definition. The subgroup findings were based on study-level data and should be considered exploratory. Finally, the estimates for the safety outcomes were imprecise. For late-onset sepsis, the pooled effect was statistically significant, but its confidence interval ranged from a small to a moderate increase in risk. The size of the excess risk, and therefore the number of infants affected in a given unit, remains uncertain. For gastrointestinal perforation, the confidence interval included both no effect and a substantial increase, so a clinically important harm cannot be excluded. Outcome definitions also differed between studies. Sepsis required culture confirmation in some studies and was defined clinically in others. Perforation was reported either as spontaneous intestinal perforation or as any gastrointestinal perforation. These limits on precision and consistency prevent a definitive statement about the balance of risk and benefit.

### 4.10 Recommendations for future research and clinical practice

Current evidence does not support the routine use of prophylactic hydrocortisone in all extremely preterm infants solely to prevent BPD. The pooled results did not show a statistically significant reduction in BPD or in mortality before discharge, or a significant improvement in survival without BPD. In clinical practice, decisions regarding early hydrocortisone should therefore remain individualized and aligned with local neonatal protocols, baseline BPD risk, gestational age, hemodynamic status, infection risk, and PDA treatment strategy. The observed increase in late-onset sepsis argues for caution, particularly in units with high baseline sepsis rates or in infants requiring prolonged invasive support. Potential gastrointestinal risk should also be considered when hydrocortisone is used near the time of indomethacin exposure.

Future research should move beyond asking whether all extremely preterm infants benefit from early hydrocortisone. The more important question is which infants, if any, derive a favorable balance of benefit and harm. Adequately powered randomized trials or individual patient data meta-analyses should evaluate treatment-effect modification by gestational age, chorioamnionitis exposure, baseline respiratory status, sex, antenatal steroid exposure, and PDA treatment. Future studies should standardize BPD definitions, report cumulative hydrocortisone exposure clearly, and prespecify late-onset sepsis, gastrointestinal perforation, and neurodevelopmental outcomes as key safety endpoints. Longer-term follow-up should include school-age cognition, executive function, behavior, growth, respiratory morbidity, hearing, and vision, rather than relying only on early developmental assessments.

## 5 Conclusion

Early prophylactic hydrocortisone did not significantly improve survival without BPD, reduce BPD at 36 weeks' postmenstrual age, or lower mortality before discharge in extremely preterm infants. Possible benefits in infants exposed to chorioamnionitis or born before 26 weeks should be considered exploratory. Hydrocortisone increased late-onset sepsis risk, with no significant differences in gastrointestinal perforation, necrotizing enterocolitis, major neonatal morbidities, or available neurodevelopmental outcomes. Current evidence does not support routine universal use; further powered trials and patient-level analyses are needed.

## Data Availability

The original contributions presented in the study are included in the article/Supplementary Material, further inquiries can be directed to the corresponding author.
