# Supplementary material for: Early prophylactic low-dose hydrocortisone for bronchopulmonary dysplasia prevention in extremely preterm infants: a systematic review and meta-analysis
Source: Front Pediatr. 2026 Oct 26;14:1934115. doi: 10.3389/fped.2026.1934115 (PMC13745721; doi:10.3389/fped.2026.1934115)
Supplement: Supplementary file 1 [file Supplementaryfile1.docx]

**Supplementary Figure 1. Leave-one-out sensitivity analysis for survival without bronchopulmonary dysplasia**

**Supplementary Figure 2. Funnel plot for survival without bronchopulmonary dysplasia**

**Supplementary Figure 3. Effect of early prophylactic hydrocortisone on duration of mechanical ventilation**

**Supplementary Figure 4. Effect of early prophylactic hydrocortisone on duration of oxygen therapy**

**Supplementary Figure 5. Effect of early prophylactic hydrocortisone on oxygen requirement at hospital discharge**

**Supplementary Figure 6. Effect of early prophylactic hydrocortisone on gastrointestinal perforation**

**Supplementary Figure 7. Effect of early prophylactic hydrocortisone on necrotizing enterocolitis**

**Supplementary Figure 8. Effect of early prophylactic hydrocortisone on late-onset sepsis**

**Supplementary Figure 9. Effect of early prophylactic hydrocortisone on hypertension requiring treatment**

**Supplementary Figure 10. Effect of early prophylactic hydrocortisone on insulin requirement**

**Supplementary Figure 11. Effect of early prophylactic hydrocortisone on medically and surgically treated patent ductus arteriosus**

**Supplementary Figure 12. Effect of early prophylactic hydrocortisone on severe intraventricular hemorrhage**

**Supplementary Figure 13. Effect of early prophylactic hydrocortisone on periventricular leukomalacia**

**Supplementary Figure 14. Effect of early prophylactic hydrocortisone on retinopathy of prematurity**

**Supplementary Figure 15. Effect of early prophylactic hydrocortisone on pulmonary hemorrhage**

**Supplementary Figure 16. Effect of early prophylactic hydrocortisone on neurodevelopmental impairment at 2 years and 5–7 years**

**Supplementary Figure 17. Effect of early prophylactic hydrocortisone on cerebral palsy at 2 years and 5–7 years**

**Supplementary Figure 18. Effect of early prophylactic hydrocortisone on cognitive impairment at 2 years and 5–7 years**

**Supplementary Figure 19. Effect of early prophylactic hydrocortisone on auditory impairment at 2 years and 5–7 years**

**Supplementary Figure 20. Effect of early prophylactic hydrocortisone on visual impairment at 2 years and 5–7 years**
