# Supplementary material for: Early prophylactic low-dose hydrocortisone for bronchopulmonary dysplasia prevention in extremely preterm infants: a systematic review and meta-analysis
Source: Front Pediatr. 2026 Oct 26;14:1934115. doi: 10.3389/fped.2026.1934115 (PMC13745721; doi:10.3389/fped.2026.1934115)
Supplement: Supplementary file 2 [file Supplementaryfile2.docx]

**Supplementary Table 1:** Detailed search strategy for each database

| Database | Search term | Filter | Results |
| --- | --- | --- | --- |
| PubMed | ("hydrocortisone" OR "cortisol" OR "glucocorticoid" OR "corticosteroid") AND ("bronchopulmonary dysplasia" OR "BPD" OR "chronic lung disease" OR "CLD") AND ("preterm" OR "premature" OR "extremely preterm" OR "very preterm" OR "extremely low birth weight" OR "ELBW" OR "very low birth weight" OR "VLBW" OR "neonate*" OR "infant*") | All Filed | 681 |
| Scopus | ("hydrocortisone" OR "cortisol" OR "glucocorticoid" OR "corticosteroid") AND ("bronchopulmonary dysplasia" OR "BPD" OR "chronic lung disease" OR "CLD") AND ("preterm" OR "premature" OR "extremely preterm" OR "very preterm" OR "extremely low birth weight" OR "ELBW" OR "very low birth weight" OR "VLBW" OR "neonate*" OR "infant*") | Article title, abstract, keywords with article limited | 2,422 |
| Web of Science | ("hydrocortisone" OR "cortisol" OR "glucocorticoid" OR "corticosteroid") AND ("bronchopulmonary dysplasia" OR "BPD" OR "chronic lung disease" OR "CLD") AND ("preterm" OR "premature" OR "extremely preterm" OR "very preterm" OR "extremely low birth weight" OR "ELBW" OR "very low birth weight" OR "VLBW" OR "neonate*" OR "infant*") | All Filed | 864 |
| Cochrane library | ("hydrocortisone" OR "cortisol" OR "glucocorticoid" OR "corticosteroid") AND ("bronchopulmonary dysplasia" OR "BPD" OR "chronic lung disease" OR "CLD") AND ("preterm" OR "premature" OR "extremely preterm" OR "very preterm" OR "extremely low birth weight" OR "ELBW" OR "very low birth weight" OR "VLBW" OR "neonate*" OR "infant*") | All Filed | 332 |

**Supplementary Table 2:**  Risk of bias of case-control studies using the Newcastle Ottawa Scale.

| **Study ID** | **Case-Control studies** | | | | |
| --- | --- | --- | --- | --- | --- |
|  | **Selection** | **Comparability** | **Exposure** | **Total score** | **Quality score** |
| Purcell et al. (2026) | **⭐⭐⭐⭐⭐** | **⭐⭐** | **⭐⭐⭐** | 9 | High |
| Yao et al. (2025) | **⭐⭐⭐⭐⭐** | **⭐⭐** | **⭐⭐⭐** | 9 | High |

**Supplementary Table 3:**  Risk of bias of Cohort studies using the Newcastle Ottawa Scale.

| **Study ID** | **Cohort studies** | | | | |
| --- | --- | --- | --- | --- | --- |
|  | **Selection** | **Comparability** | **Outcome** | **Total score** | **Quality score** |
| Smedbäck et al. (2026) | **⭐⭐⭐⭐⭐** | **⭐⭐** | **⭐⭐⭐** | 9 | High |
| Shah et al. (2024) | **⭐⭐⭐⭐⭐** | **⭐** | **⭐⭐⭐** | 8 | High |
